# Development and validation of a nomogram for patients with stage II/III gastric adenocarcinoma after radical surgery

**DOI:** 10.3389/fsurg.2022.956256

**Published:** 2022-10-31

**Authors:** Lei Wang, Huiqiong Han, Liwen Feng, Yanru Qin

**Affiliations:** Department of Oncology, The First Affiliated Hospital of Zhengzhou University, Zhengzhou, China

**Keywords:** nomogram, gastric adenocarcinoma, decision curve analysis, prognosis, survival, plateletalbumin ratio

## Abstract

**Background:**

We aimed to construct nomograms based on clinicopathological features and routine preoperative hematological indices to predict cancer-specific survival (CSS) and disease-free survival (DFS) in patients with stage II/III gastric adenocarcinoma (GA) after radical resection.

**Methods:**

We retrospectively analyzed 468 patients with stage II/III GA after curative gastrectomy between 2012 and 2018; 70% of the patients were randomly assigned to the training set (*n* = 327) and the rest were assigned to the validation set (*n* = 141). The nomogram was constructed from independent predictors derived from the Cox regression in the training set. Using the consistency index, the calibration and the time-dependent receiver operating characteristic curves were used to evaluate the accuracy of the nomogram. Decision curve analysis was used to assess the value of the model in clinical applications. Patients were further divided into low- and high-risk groups based on the nomogram risk score.

**Results:**

Multivariate Cox model identified depth of invasion, lymph node invasion, tumor differentiation, adjuvant chemotherapy, CA724, and platelet-albumin ratio as covariates associated with CSS and DFS. CA199 is a risk factor unique to CSS. The nomogram constructed using the results of the multivariate analysis showed high accuracy with a consistency index of 0.771 (CSS) and 0.771 (DFS). Moreover, the area under the curve values for the 3-and 5-year CSS were 0.868 and 0.918, and the corresponding values for DFS were 0.872 and 0.919, respectively. The nomogram had a greater clinical benefit than the TNM staging system. High-risk patients based on the nomogram had a worse prognosis than low-risk patients.

**Conclusion:**

The prognostic nomogram for patients with stage II/III GA after radical gastrectomy established in this study has a good predictive ability, which is helpful for doctors to accurately evaluate the prognosis of patients to make more reasonable treatment plans.

## Introduction

Gastric adenocarcinoma (GA), which accounts for 95% of gastric malignancies, is the fifth most common cancer and a major global health challenge (1). GA usually originates from the lining of the stomach, and its early symptoms are not obvious; therefore, many patients with GA are not diagnosed until the metastatic or advanced stage (2). Radical surgery with subsequent adjuvant chemotherapy is the main treatment for early stage GA; however, the overall survival rate remains poor due to the high frequency of metastasis and recurrence (3). Therefore, there is an urgent need to individually analyze independent risk factors and establish novel predictive models that can accurately identify high-risk patients with GA.

Multiple studies have shown that nutritional factors, inflammation, and coagulation are associated with cancer patient outcomes, including GA (4–6). Systemic inflammation and immune evasion are the cardinal features of malignancy, and various inflammatory factors contribute to tumor progression. Interestingly, in addition to neutrophils and lymphocytes, recent studies have revealed that platelets are potent immune modulators and effectors, including direct identification and elimination of pathogens or enhancement of leukocyte immunity, in addition to their central role in hemostasis (7). Additionally, nutritional status is also a critical part of cancer management, especially in gastrointestinal tumors with high prevalence of malnutrition (8). Feng et al. reported that lower preoperative serum albumin levels are associated with unfavorable prognosis in patients with gastric cancer (9). Furthermore, patients with cancer often have abnormal coagulation, which is closely related to tumor progression (10). Several peripheral blood markers have been shown to correlate with cancer prognosis, including the platelet-albumin ratio (PAR), platelet-lymphocyte ratio (PLR), albumin-fibrinogen ratio (AFR), lymphocyte-monocyte ratio (LMR), neutrophil-lymphocyte ratio (NLR), aspartate aminotransferase-alanine aminotransferase ratio (SLR), and D-dimer (9, 11). These indicators can directly reflect inflammation, nutritional level, liver function, and coagulation in patients with cancer. In addition, some classic tumor markers, including carcinoembryonic antigen (CEA), carbohydrate antigen 19-9 (CA19-9), and CA72-4, are widely used for prognostication of cancer patients (12). The hematological indicators mentioned above from routine testing are economical and readily available; therefore, we selected these indicators for evaluation as potential predictive factors.

The nomogram can visualize and integrate independent predictors, realize individualized prognostic assessment, and improve accuracy, and has been studied and applied to multiple cancer types (13, 14). In this study, we attempted to combine clinicopathological characteristics and preoperative routine laboratory indicators to construct and verify a nomogram for patients with stage II/III GA after radical surgery. This personalized prediction system can facilitate clinicians in identifying high-risk patients to develop more personalized treatments.

## Materials and methods

### Patients and data collection

We retrospectively collected the data of 468 patients with stage II/III GA who underwent radical gastrectomy at the First Affiliated Hospital of Zhengzhou University between May 2012 and May 2018. Seventy percent of the patients were randomly selected as the training set (*n* = 327), and the rest were assigned to the validation set (*n* = 141). The inclusion criteria were as follows: (1) histologically confirmed stage II/III GA; (2) R0 resection; (3) no antitumor therapy before surgery; (4) complete clinicopathological and follow-up data, and all hematological parameters to be assessed should be measured within 1 week before surgery; (5) no other malignancies; (6) no parenteral nutrition, acute inflammation, or significant organ damage within 1 week before surgery; and (7) no cause of death other than GA.

Widely accepted thresholds for grouping continuous variables: D-Dimer (0.3 mg/L), CA199 (35 U/ml), CA724 (6.9 U/ml), and CEA (5 U/ml). The optimal cutoff values for age (68), tumor size (3.5 cm), PAR (6.4), AFR (9.7), NLR (2.3), LMR (3.1), SLR (1.8), and PLR (202.9) were determined using X-tile (15) analysis because of the lack of a defined threshold. Studies involving human participants were reviewed and approved by the Medical Ethics Committee of the First Affiliated Hospital of Zhengzhou University. According to the Declaration of Helsinki, patient data were anonymized and kept confidential. Due to the retrospective nature of this study, informed consent was not obtained.

### Follow-up and outcome

Follow-up was done *via* different methods such as medical records and telephone surveys. Patients were observed after curative gastrectomy every 3 months during the first year, every 6 months for 2–3 years, and annually thereafter for up to 5 years post-surgery. Each follow-up included physical examination, laboratory testing, electronic gastroscopy, as clinically indicated, and chest/abdomen/pelvic enhanced computed tomography. The primary endpoint of this study was cancer-specific survival (CSS), which was defined as the time from the date of surgery to cancer-related death. Disease-free survival (DFS) was defined as the time from curative surgery to death, recurrence, or the final follow-up.

### Statistical analysis

Statistical analysis was performed using SPSS Statistics (version 26.0, IBM, USA) and R software (version 4.1.2). The optimal cut-off value was determined using X-tile software. The continuous variables were transformed into categorical variables. The chi-square test was used to compare categorical data. Statistical significance was set at *P* < 0.05. For continuous variables, the Kolmogorov–Smirnov test was first performed. If an approximately normal distribution was displayed, the data were described using the mean and standard deviation. Otherwise, the median with the interquartile range (IQR) was used. First, the proportional hazards hypothesis test was performed using the Cox regression model. If the hypothesis was not satisfied, a Cox regression model with time-dependent covariates was used (16). Significant factors in the univariate analysis will be included in the Cox regression equation to identify independent factors that will be used to construct nomograms. Nomograms for CSS and DFS were constructed using the rms and survival packages in the R software. Internal validation was performed to demonstrate the reliability and repeatability of the nomograms. The consistency index (C-index), calibration curve, and time-dependent receiver operating characteristic curve were used to evaluate the accuracy and discriminative ability of the prediction map in the training and validation sets, respectively. The ggDCA package in the R software was used to construct the decision curve analysis (DCA) to further evaluate the clinical benefit of the nomogram. Each patient was scored using the survival package in R software and divided into high- and low-risk groups based on the median risk score. The Kaplan–Meier method was used to draw the CSS and DFS survival curves, and the log-rank test was used for statistical analysis.

## Results

### Clinicopathological characteristics

Table 1 shows the clinicopathological characteristics of the 468 patients with GA, including 327 patients in the training set and 141 patients in the validation set. In the training set, the age of the patients at diagnosis ranged from 25 to 88 years, with a median age of 61 years (IQR, 52–66 years). Most patients were male (*n* = 240, 73.4%), and the rest were female (*n* = 87, 26.6%). The median tumor size was 4.5 cm (IQR, 3.5–6.0 cm). According to the 8th edition of the American Joint Committee on Cancer staging system, it is more common in patients with pT3–4 (*n* = 247, 75.5%), and most patients have lymph node invasion (*n* = 244, 74.6%). Most patients with GA were classified as stage III (*n* = 214, 65.4%). Poor differentiation (*n* = 257, 78.6%) was the most common tumor grade. In total, 184 patients (56.3%) received adjuvant chemotherapy. Furthermore, the baseline characteristics did not differ between the training and validation groups (*P* > 0.05). For all patients, the final follow-up period ranged from 0.5 to 96 months, with a median of 36.0 months. In the training set, the 1-, 3-, and 5-year CSS rates were 82.9, 48.6, and 40.3%, respectively. The 1-, 3-, and 5-year DFS rates were 75.5, 46.8, and 39.5%, respectively. In the validation set, the 1-, 3-, and 5-year CSS rates were 81.6, 51.1, and 44.6%, respectively. The 1-, 3-, and 5-year DFS rates were 75.2, 48.9, and 42.9%, respectively.

**Table 1:** Baseline characteristics of patients in the training and validation cohorts.

Variables	NO. (%)		X^2^	*P*
	Training Cohort (*n* = 327)	Validation Cohort (*n* = 141)		
Sex			0.5	0.480
Male	240 (73.4)	99 (70.2)		
Female	87 (26.6)	42 (29.8)		
Age, years			3.754	0.053
≤68	263 (80.4)	102 (72.3%)		
>68	64 (19.6)	39 (27.7)		
Family history			0.41	0.522
No	268 (82.0)	119 (84.5)		
Yes	59 (18.0)	22 (15.5)		
Diabetes			0.653	0.419
No	312 (95.4)	132 (93.6)		
Yes	15 (4.6)	9 (6.4)		
Hypertension			0.044	0.843
No	264 (80.7)	115 (81.6)		
Yes	63 (19.3)	26 (18.4)		
Tobacco			0.076	0.783
No	236 (72.2)	100 (70.9)		
Yes	91 (27.8)	41 (29.1)		
Alcohol			0.217	0.641
No	263 (80.4)	116 (82.3)		
Yes	64 (19.6)	25 (17.7)		
Depth of invasion			2.041	0.153
T1-2	80 (24.5)	26 (18.4)		
T3-4	247 (75.5)	115 (81.6)		
Lymph node invasion			0.126	0.722
N0	83 (25.4)	38 (27.0)		
N1-3	244 (74.6)	103 (73.0)		
TNM stage			0.066	0.798
II	113 (34.6)	47 (33.3)		
III	214 (65.4)	94 (66.7)		
Tumor differentiation			0.661	0.416
Middle or high	70 (21.4)	35 (24.8)		
low	257 (78.6)	106 (75.2)		
Size, cm			0.247	0.619
≤3.5	105 (32.1)	42 (29.8)		
>3.5	222 (67.9)	99 (70.2)		
Tumor location			4.5	0.104
Upper 1/3	166 (50.8)	75 (53.2)		
Middle 1/3	73 (22.3)	20 (14.2)		
Lower 1/3	88 (26.9)	46 (32.6)		
Adjuvant chemotherapy			0.002	0.962
No	143 (43.7)	62 (44.0)		
Yes	184 (56.3)	79 (56.0)		
CA199, U/ml			0.306	0.580
≤35	257 (78.6)	114 (80.9)		
>35	70 (21.4)	27 (19.1)		
CA724, U/ml			0.017	0.898
≤6.9	251 (76.8)	109 (77.3)		
>6.9	76 (23.2)	32 (22.7)		
CEA, U/ml			0.229	0.632
≤5	257 (78.6)	108 (76.6)		
>5	70 (21.4)	33 (23.4)		
D-Dimer, mg/l			0.065	0.798
≤0.3	238 (74.3)	101 (71.6)		
>0.3	89 (27.2)	40 (28.4)		
PLR			0.514	0.473
≤202.9	245 (74.9)	110 (78.0)		
>202.9	82 (25.1)	31 (22.0)		
PAR			1.515	0.218
≤6.4	177 (54.1)	85 (60.3)		
>6.4	150 (45.9)	56 (39.7)		
AFR			0.181	0.671
<9.7	49 (15.0)	19 (13.5)		
≥9.7	278 (85.0)	122 (86.5)		
NLR			0.224	0.636
≤2.3	184 (56.3)	76 (53.9)		
>2.3	143 (43.7)	65 (46.1)		
LMR			0.061	0.805
<3.1	119 (36.4)	53 (37.6)		
≥3.1	208 (63.6)	88 (62.4)		
SLR			0.119	0.731
≤1.8	280 (85.6)	119 (84.4)		
>1.8	47 (14.4)	22 (15.6)		

### Independent predictors

Tables 2, 3 show the results of univariate and multivariate Cox analyses on the training set data, including hazard ratios and 95% confidence intervals (CI). Univariate analysis showed that age, depth of invasion, lymph node invasion, tumor size, differentiation, adjuvant chemotherapy, CA199, CA724, D-dimer, PLR, PAR, SLR, and AFR were related to CSS and DFS, while CEA was only relevant for DFS (*P* < 0.05). Significant factors in the univariate analysis were included in multivariate analysis. Depth of invasion (*P* < 0.001), lymph node invasion (*P* < 0.001), tumor differentiation (*P* = 0.015), adjuvant chemotherapy (*P* = 0.001), CA199 (*P* = 0.038), CA724 (*P* = 0.002), and PAR (*P* = 0.001) were independent predictors of CSS, while depth of invasion (*P* < 0.001), lymph node invasion (*P* < 0.001), tumor differentiation (*P* = 0.003), adjuvant chemotherapy (*P* = 0.004), CA724 (*P* = 0.001), and PAR (*P* = 0.002) were independent predictors of DFS.

**Table 2:** Univariate and multivariate cox regression analyses of prognostic factors for cancer-specific survival.

Variables	Univariate Analysis		Multivariate Analysis	
	HR (95% CI)	*P*-value	HR (95% CI)	*P*-value
Sex (male)	0.929 (0.674–1.283)	0.656		
Age (>68)	1.721 (1.239–2.390)	**0** **.** **001**	0.953 (0.660–1.377)	0.797
Family history (yes)	0.959 (0.659–1.398)	0.829		
Diabetes (yes)	1.458 (0.793–2.681)	0.225		
Hypertension (yes)	1.000 (0.695–1.439)	0.999		
Tobacco (yes)	0.750 (0.536–1.049)	0.093		
Alcohol (yes)	0.720 (0.487–1.065)	0.100		
Depth of invasion (T3-4)	4.981 (2.979–8.326)	**<0** **.** **001**	6.495 (3.763–11.208)	**<0** **.** **001**
Lymph node invasion (N1-3)	3.111 (2.037–4.753)	**<0** **.** **001**	4.762 (2.995–7.573)	**<0** **.** **001**
Differentiation (low)	1.720 (1.169–2.531)	**0** **.** **005**	1.657 (1.102–2.492)	**0** **.** **015**
Tumor size (>3.5)	1.852 (1.327–2.585)	**<0** **.** **001**	1.042 (0.731–1.486)	0.819
Tumor location				
Upper 1/3	1			
Middle 1/3	1.102 (0.771–1.574)	0.515		
Lower 1/3	0.853 (0.601–1.212)	0.375		
Adjuvant chemotherapy (yes)	0.599 (0.449–0.799)	**<0** **.** **001**	0.582 (0.426–0.796)	**0** **.** **001**
CA199 (>35)	2.385 (1.740–3.269)	**<0** **.** **001**	1.436 (1.019–2.023)	**0** **.** **038**
CA724 (>6.9)	2.800 (2.056–3.812)	**<0** **.** **001**	1.704 (1.223–2.375)	**0** **.** **002**
CEA (>5)	1.362 (0.974–1.906)	0.071		
D-Dimer (>0.3)	1.575 (1.160–2.139)	**0** **.** **004**	1.165 (0.843–1.609)	0.355
PLR (>202.9)	1.839 (1.355–2.496)	**<0** **.** **001**	0.878 (0.620–1.242)	0.461
PAR (>6.4)	2.660 (1.980–3.575)	**<0** **.** **001**	1.822 (1.299–2.556)	**0** **.** **001**
AFR (<9.7)	2.469 (1.741–3.500)	**<0** **.** **001**	1.081 (0.734–1.592)	0.693
NLR (>2.3)	0.814 (0.608–1.090)	0.168		
LMR (<3.1)	0.745 (0.548–1.014)	0.061		
SLR (>1.8)	1.728 (1.194–2.499)	**0** **.** **004**	1.010 (0.682–1.498)	0.959

**Table 3:** Univariate and multivariate cox regression analyses of prognostic factors for disease-free survival.

Variables	Univariate Analysis		Multivariate Analysis	
	HR (95% CI)	*P*-value	HR (95% CI)	*P*-value
Sex (male)	0.926 (0.675–1.271)	0.636		
Age (>68)	1.631 (1.78–1.2.258)	**0** **.** **003**	0.930 (0.648–1.336)	0.695
Family history (yes)	0.952 (0.657–1.379)	0.793		
Diabetes (yes)	1.359 (0.739–2.499)	0.323		
Hypertension (yes)	1.074 (0.755–1.526)	0.692		
Tobacco (yes)	0.719 (0.489–1.056)	0.093		
Alcohol (yes)	0.718 (0.488–1.055)	0.091		
Depth of invasion (T3-4)	4.265 (2.652–6.858)	**<0** **.** **001**	5.951 (3.583–9.886)	**<0** **.** **001**
Lymph node invasion (N1-3)	3.180 (2.096–4.824)	**<0** **.** **001**	4.848 (3.067–7.662)	**<0** **.** **001**
Differentiation (low)	1.712 (1.175–2.493)	**0** **.** **005**	1.887 (1.241–2.870)	**0** **.** **003**
Tumor size (>3.5)	1.908 (1.372–2.654)	**<0** **.** **001**	1.091 (0.767–1.551)	0.630
Tumor location				
Upper 1/3	1			
Middle 1/3	1.147 (0.809–1.627)	0.442		
Lower 1/3	0.831 (0.586–1.179)	0.300		
Adjuvant chemotherapy (yes)	0.650 (0.489–0.863)	**0** **.** **003**	0.642 (0.473–0.870)	**0** **.** **004**
CA199 (>35)	2.426 (1.776–3.315)	**<0** **.** **001**	1.315 (0.924–1.871)	0.128
CA724 (>6.9)	2.905 (2.138–3.948)	**<0** **.** **001**	1.735 (1.235–2.438)	**0** **.** **001**
CEA (>5)	1.424 (1.024–1.979)	**0** **.** **036**	1.390 (0.953–2.029)	0.087
D-Dimer (>0.3)	1.473 (1.086–1.996)	**0** **.** **013**	1.065 (0.768–1.476)	0.706
PLR (>202.9)	1.863 (1.378–2.519)	**<0** **.** **001**	0.964 (0.684-1.359	0.834
PAR (>6.4)	2.542 (1.899–3.401)	**<0** **.** **001**	1.718 (1.226–2.407)	**0** **.** **002**
AFR (<9.7)	2.356 (1.6663–3.338)	**<0** **.** **001**	1.097 (0.753–1.599)	0.628
NLR (>2.3)	0.808 (0.605–1.078)	0.147		
LMR (<3.1)	0.764 (0.564–1.035)	0.082		
SLR (>1.8)	1.750 (1.216–2.518)	**0** **.** **003**	1.041 (0.708–1.529)	0.839

### Construction and verification of nomogram models

**Figure 1:**
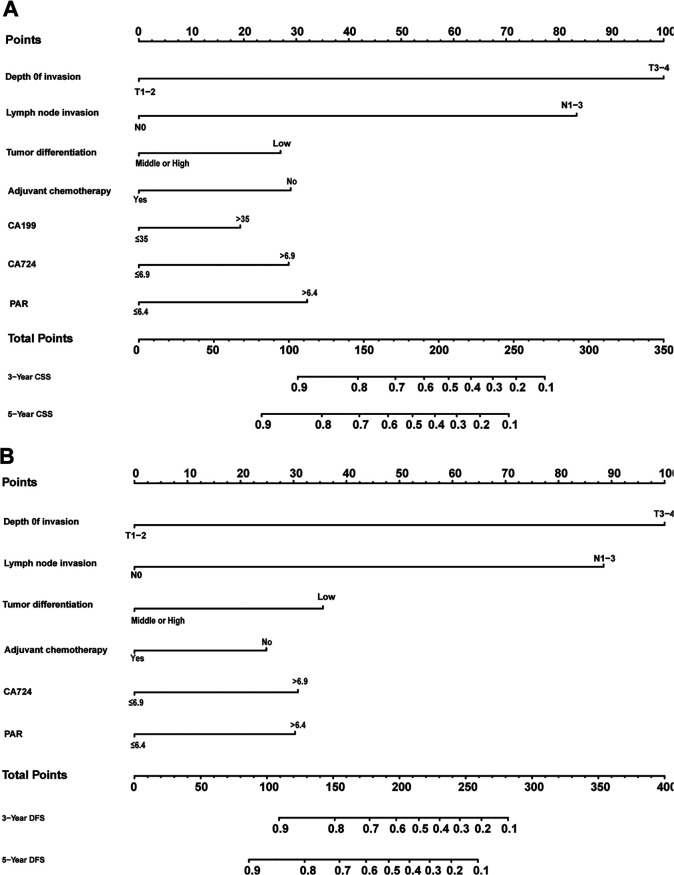
Nomogram predicting cancer-specific survival (**A**) and disease-free survival (**B**) of patients with stage II/III gastric adenocarcinoma who underwent gastrectomy.

**Figure 2:**
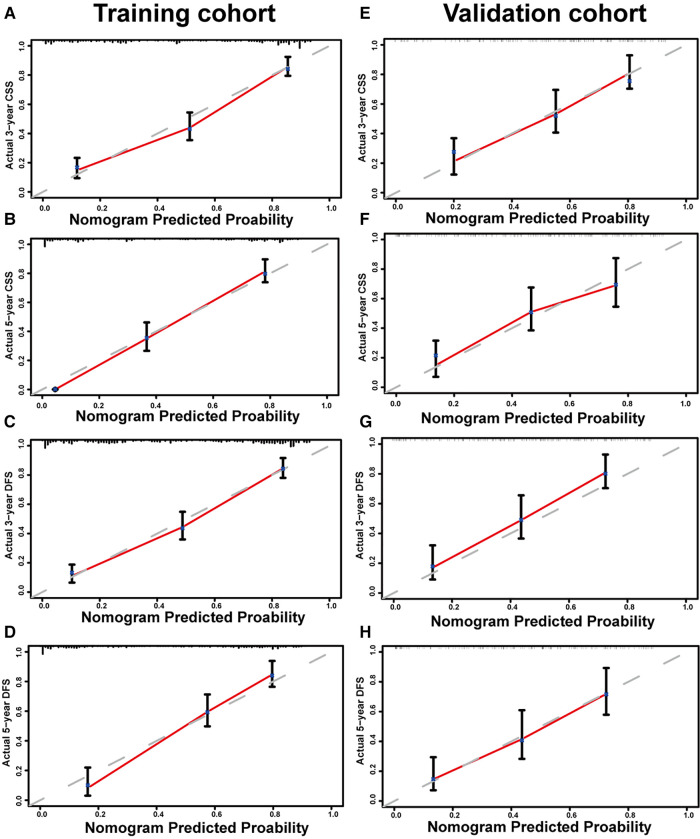
Calibration curves of nomogram for predicting 3-year cancer-specific survival (CSS) (**A**), 5-year CSS (**B**), 3-year disease-free survival (DFS) (**C**), and 5-year DFS (**D**) in the training set. Calibration curves of nomogram for predicting 3-year CSS (**E**), 5-year CSS (**F**), 3-year DFS (**G**), and 5-year DFS (**H**) in the validation set.

**Figure 3:**
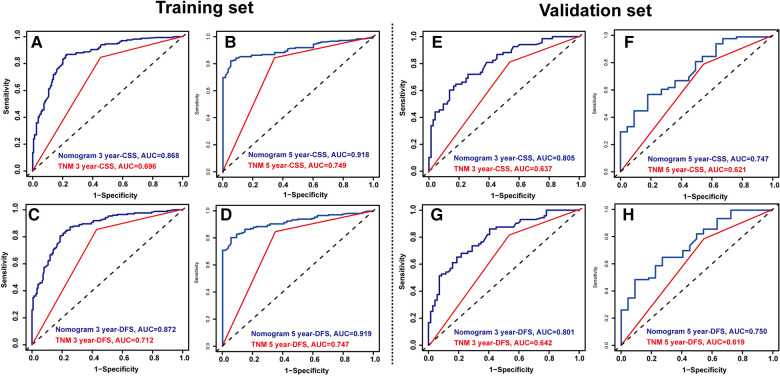
Time-dependent reciever operating characteristics (ROC) curves of nomograms and TNM staging were used to test the predictive power of the 3-year cancer-specific survival (CSS) (**A**) and 5-year CSS (**B**), 3- year disease-free survival (DFS) (**C**) and 5-year DFS (**D**) in the training set. Time-dependent ROC curves of nomograms and TNM staging were used to test the predictive power of the 3-year CSS (**E**) and 5-year CSS (**F**), 3- year DFS (**G**) and 5-year DFS (**H**) in the validation set.

Figure 1 shows the nomogram predicting CSS and DFS that was constructed based on the results of the multivariate analysis with hazard ratios. The models can score each patient, and the higher the score, the worse is the prognosis. The C-index for predicting CSS and DFS was 0.771 (95%CI, 0.738–0.804) and 0.771 (95%CI, 0.740–0.802), suggesting that the constructed nomogram had an accurate predictive ability. In addition, as shown in Figure 2, the calibration curve is close to the diagonal line, suggesting that the nomogram predicts the patients' 3- and 5-year CSS and DFS to be similar to the actual situation, further illustrating the predictive accuracy of the model. In the nomogram model, the area under the curve (AUC) for the 3- and 5-year CSS were 0.868 (95%CI, 0.829–0.907) and 0.918 (95%CI, 0.884–0.953), respectively, and the AUC for the 3- and 5-year DFS was 0.872 (95%CI, 0.834–0.910) and 0.919 (95%CI, 0.885–0.952), respectively. In TNM staging, the AUC for 3- and 5-year CSS were 0.696 (95%CI, 0.648–0.743) and 0.749 (95%CI, 0.683–0.816), respectively, and the AUC for 3- and 5-year DFS were 0.712 (95%CI, 0.664–0.760) and 0.747 (95%CI, 0.678–0.816), respectively. These results showed that the nomogram model had a higher accuracy than TNM staging (Figure 3). Similarly, in the validation set, the C-indices for CSS and DFS were 0.734 (95%CI, 0.679–0.789) and 0.731 (95%CI, 0.680–0.782), respectively, and the calibration curve predicted survival probability with high agreement, which also proved the reliability of the model.

### Decision curve analysis and survival curves based on the nomograms

**Figure 4:**
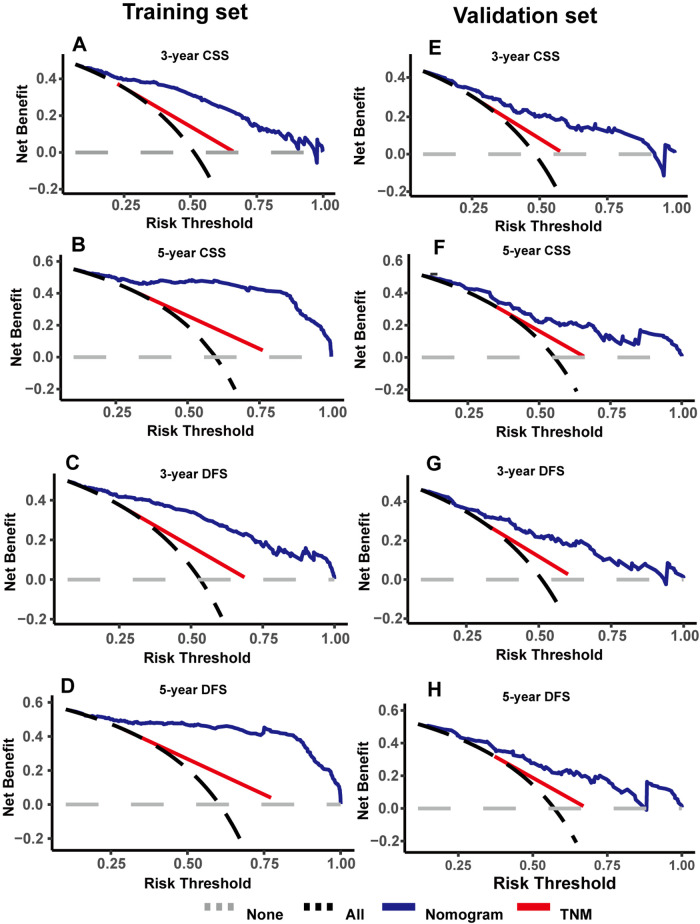
Decision curve analysis were used to compare the clinical benefit of nomogram and TNM staging in cancer-specific survival (CSS) (**A,B**) and disease-free survival (DFS) (**C,D**) in the training set; decision curve analysis were used to compare the clinical benefit of nomogram and TNM staging in CSS (**E,F**) and DFS (**G,H**) in the validation set.

**Figure 5:**
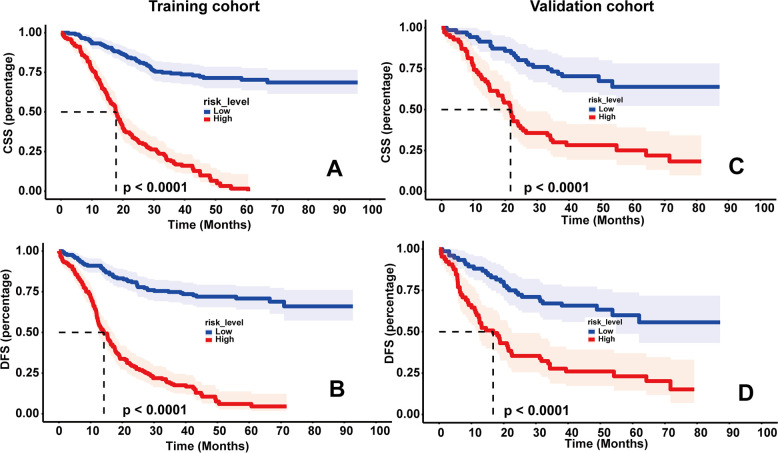
Kaplan–meier survival curves of cancer-specific survival (CSS) (**A**) and disease-free survival (DFS) (**B**) in the training set based on risk scores; kaplan–meier survival curves of CSS (**C**) and DFS (**D**) in the validation set based on risk scores.

As shown in Figure 4, DCA was used to analyze the net benefit rate of the nomogram and TNM staging at different threshold probabilities. The results showed that the nomogram had a higher clinical benefit than the TNM staging system when the probability threshold was 0.2 to 0.8, whether it was 3-year or 5-year CSS, or 3-year or 5-year DFS. The results of the validation set were consistent with those of the training set. Figure 5 shows that patients in the high-risk group, based on the median nomogram score, had a worse prognosis than those in the low-rank group (*P* < 0.001).

## Discussion

GA is a complex disease and surgical or endoscopic resection remains the only cure. However, the current survival rate of patients with GA remains low, even with the combined efforts of multidisciplinary teams. Over the years, tumor microenvironment has been the focus of our research (17, 18), and chronic inflammation and immune dysfunction are its most important features (19). The release of various pro-inflammatory cytokines and inflammatory substances such as interleukin-1β, interleukin-8, and tumor necrosis factor-α promotes GA progression (19). Neutrophils and lymphocytes are the most important immune cells that can intuitively reflect the level of inflammation in the human body, and abnormal changes in these cells are thought to be related to tumor progression. Recently, in addition to their known hemostatic effects, researchers found that platelets are potent immunomodulators that play an important role in regulating systemic inflammation and immunity (20). Moreover, tumors release a variety of cytokines including vascular endothelial growth factor, platelet-derived growth factor, and transforming growth factor-β1 that can promote platelet production, and the increased platelets can shield tumor cells in peripheral blood and interfere with immune cells to enhance the metastatic potential of tumors (21, 22). However, the nutritional status of cancer patients is an important part of cancer management. The prevalence of malnutrition in patients with cancer is high, particularly in those with gastrointestinal tumors. According to one study, the prevalence of malnutrition in digestive malignancies is approximately 52% (23). Albumin is widely recognized as an indicator of nutritional levels, and the mechanism of hypoalbuminemia is related to the increase in two inflammatory cytokines, tumor necrosis factor-α and interleukin-6, which inhibit the synthesis of albumin (24). A study by Feng et al. indicated that malnutrition is significantly associated with poor prognosis in patients with gastric cancer (9). Hypercoagulability is an important physiological characteristic of patients with malignant tumors. Abnormalities in indicators that can represent coagulation, such as D-dimer and fibrinogen, are often associated with poor prognosis in patients with tumors (25, 26).

In our study, we mainly focused on PLR, PAR, NLR, LMR, STR, AFR, and D-dimer levels, which can reflect inflammation, nutritional status, liver function, and coagulation function in patients. These routine hematological markers are readily available and economical, and previous studies have shown that these indicators are associated with the prognosis of malignancies (11). The NLR and LMR are indicators of systemic inflammation. Previous studies have shown that an increase in NLR or decrease in LMR indicates that the inflammatory response promotes tumor development, indicating a poor prognosis for patients (27, 28). The relationship between PLR and cancer prognosis is unclear, and the underlying mechanism may be related to platelet and lymphocyte functions (28). SLR is an indicator of liver function. Previous studies have indicated that people with an elevated SLR have an increased risk of gastric cancer (29). PAR has also been explored in a variety of other cancers, such as pancreatic, esophageal, and liver cancers; however, its significance in GA has not been explored (30, 31). The study of Huang et al. pointed out that PAR is an independent predictor of esophageal squamous cell carcinoma and can accurately predict the prognosis of patients with this cancer (32). Similar to PAR, AFR also reflects multiple patient metrics, including nutritional levels and coagulation. Feng et al. confirmed its association with gastric cancer prognosis (9). A lower AFR appears to indicate a worse prognosis. In our study, compared to PAR, the other remaining indicators showed no statistical significance as independent prognostic factors in the multivariable Cox regression analysis. This result suggests that we may need to pay more attention to the interaction of platelets with tumor cells and other immune cells. However, preoperative attention to the nutritional status of patients and timely intervention may help prolong patient survival. A study by Bang et al. showed that postoperative platinum-based chemotherapy in patients with gastric cancer can effectively prolong the survival time of patients (33). Our study is consistent with previous findings that timely adjuvant chemotherapy is one of the protective factors for patient outcomes.

A nomogram is an effective predictive tool that quantifies individual risk, and its intuitive and visual features make predictive models more readable and facilitate clinical application (13). In this study, we constructed a nomogram to predict the prognosis of patients with stage II/III GA after curative gastrectomy. The results showed that TNM stage, tumor differentiation, postoperative adjuvant chemotherapy, tumor markers, and PAR were independent patient predictors. In addition, high-risk patients identified using the model had a poorer prognosis. Therefore, timely interventions for high-risk patients, including improved nutrition and inhibition of inflammation, may improve patient outcomes. Compared with the nomogram for stage II/III gastric cancer patients after curative gastrectomy followed by adjuvant chemotherapy constructed by Li-tong Shi (11), our model is different; we only focused on one pathological type, GA; we included adjuvant chemotherapy as a variable and incorporated different hematological indicators while constructing receiver operator characteristic and DCA curves to evaluate the clinical applicability of the model. In our study, the relationship between PAR and GA prognosis was demonstrated, which has not been previously reported. This further confirms that immunity and nutritional status are closely related to GA prognosis. However, we acknowledge that this study has some limitations. First, this was a retrospective single-center study, and the preliminary results need to be further validated in prospective clinical trials. Second, we only performed internal validation of the nomogram, and external validation was required to extend the applicability of the model. Third, we mainly focused on economical and convenient routine laboratory tests; therefore, we did not conduct research on EGFR mutations, Her-2 expression, ERBB2 expression, or microsatellite instability status. Some indicators, such as *Helicobacter pylori* and PG I/II, were not routinely detected; therefore, they were not included in this study. These metrics will be the focus of future research.

## Conclusion

In conclusion, the nomogram constructed in this study for patients with stage II/III GA after curative gastrectomy could accurately predict CSS and DFS. In addition, our models are more accurate than traditional TNM staging for predicting GA prognosis and may bring more clinical benefits to patients. For patients with poor outcomes, shortening the follow-up time and timely intervention can be used to prevent the occurrence of adverse events.

## Data Availability

The raw data supporting the conclusions of this article will be made available by the authors, without undue reservation.
